# Parasitic surveillance in wolves of central Italy: a focus on the Abruzzo region

**DOI:** 10.1186/s13071-026-07305-4

**Published:** 2026-02-21

**Authors:** Sabrina Vanessa Patrizia Defourny, Mariasole Colombo, Gianluca D’Amico, Stefania Salucci, Antonio Cocco, Maria Chiara Cantelmi, Daniela Averaimo, Marco Rulli, Gianfranco Romeo, Susanna Tora, Marina Baffoni, Nicola De Dominicis, Nicola D’Alterio, Antonio Petrini

**Affiliations:** 1 Istituto Zooprofilattico Sperimentale dell’Abruzzo e del Molise “G. Caporale”, Teramo, Italy; 2 University of Agricultural Sciences and Veterinary Medicine of Cluj-Napoca, Cluj-Napoca, Romania

**Keywords:** Wolf, Wildlife, Endoparasites, *Leishmania* spp., ZoonosesZoonoses

## Abstract

**Background:**

Monitoring parasitic infections in wildlife is essential for assessing ecosystem health and pathogen dynamics, particularly in apex predators like the wolf (*Canis lupus*). As top-level carnivores with wide-ranging habitats and diverse interactions with prey, wolves can serve as effective sentinels for the circulation of parasitic agents within ecosystems. The present study aimed at monitoring parasitic infections, particularly those of zoonotic importance, in wolves from central Italy.

**Methods:**

The study was conducted on 169 wolf carcasses recovered between 2018 and 2024. *Leishmania* spp. and *Trichinella* spp. infections were evaluated using molecular techniques (on 169 spleen and 150 striated muscle samples, respectively), while intestinal and extra-intestinal parasites were investigated using flotation and Baermann tests (147 fecal samples).

**Results:**

Of the 169 wolves included in the study, 89 (52.66%) were males and 131 (77.51%) were adults. Of all analyzed carcasses, 135 (80%) tested positive for ≥ 1 parasite. *Leishmania* spp. were detected in 12 of 169 (7.1%) wolves and *Trichinella* spp. were detected in 43 of 150 (28.7%) wolves. Copromicroscopic examinations revealed infections with nematodes belonging to family* Ancylostomatidae* (62%), *Trichuris vulpis* (32%), *Capillaria* spp. (20.4%), helminths from Class Cestoda (8.8%), *Angiostrongylus vasorum* (7.5%), coccidian oocysts (4.8%), *Crenosoma vulpis* (2.7%) and *Toxocara canis* (2%).

**Conclusions:**

The present study included the largest number of wolves when compared with previous similar parasitological surveys conducted in Europe. High parasite circulation shared by dogs and humans within the wolf population can be surmised, highlighting the potential role of wolves in pathogen transmission. Given their position at the wildlife/domestic animal/human interface, continuous parasitological surveillance is important not only for conservation efforts but also for protecting public and veterinary health.

**Graphical abstract:**

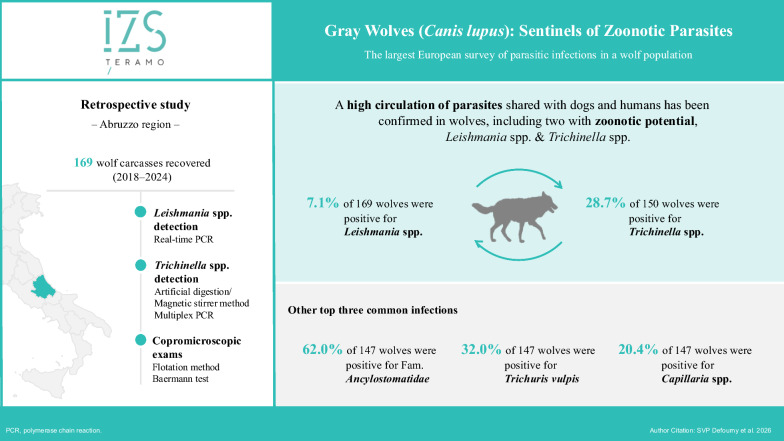

## Background

Parasites of wildlife play a critical role in ecosystem dynamics and can serve as indicators of environmental health. Monitoring infections in apex predators such as the wolf (*Canis lupus*) is particularly important, as wolves can harbor and spread a wide range of parasites, including those with zoonotic potential. Given their wide-ranging behavior and trophic position, wolves may act as both reservoirs and sentinels for emerging threats with possible public health implications.

Over past centuries, anthropogenic pressures, including hunting and the use of traps and poisoned baits, combined with deforestation and a decline in natural prey availability, have contributed to the decline of wolf populations across Europe [1]. The legal protection granted to wolves in Italy since 1976, combined with ecological changes in mountainous regions (e.g. rural depopulation, natural reforestation and the recovery of wild ungulate populations), has facilitated wolf recolonization of the Apennine ridge [1]. Simultaneously, the expansion of human activities into natural environments has intensified conflicts, particularly those due to livestock predation, and has increased interactions among wolves, domestic animals and humans, thereby increasing the potential for pathogen transmission [2–5].

Wolves can be infected by a wide range of parasitic species, many of which are shared with domestic dogs (*Canis lupus familiaris*) and humans. Among these, the protozoan *Leishmania infantum* (Kinetoplastea: Trypanosomatidae) is of particular importance*.* This parasite, transmitted through the bite of infected sandflies (*Phlebotomus* spp.), is globally distributed and infects a wide range of vertebrate hosts. Its zoonotic potential is well recognized, and the growing incidence of *L. infantum* infection in humans underscores the need for ongoing monitoring to prevent further spread. In animals, *L. infantum* is recognized as the causative agent of canine leishmaniosis, a disease widely spread in the Mediterranean basin that can present with clinical manifestations ranging from subclinical to potentially severe and fatal. *Leishmania infantum* has historically been endemic in southern Europe, but a northward expansion of the parasite and its vectors has been documented over the past decades [6–8]. Although dogs are the main reservoir for this protozoan in the Mediterranean basin, with seroprevalence up to 30%, the presence of reservoirs among wild vertebrates has been suggested as a possible factor in the lack of successful control measures [6, 9].

The authors of previous studies have reported variable positivity rates of *L. infantum* in foxes, wolves, wildcats, badgers, rats, roe deer and wild boars [6, 10–12]. To date, only a few studies have been conducted in Italy focusing on the epidemiological role of wild hosts in the persistence of *Leishmania* spp. infections in endemic areas, and most of these have focused on the red fox population [13–16]. Due to their ecological characteristics and close evolutionary relationship to domestic dogs, wolves have recently attracted attention as potential wildlife reservoirs of *L. infantum* . However, epidemiological studies on leishmaniosis in wolves in Europe are scarce [17].

In addition, wolves can host a broad spectrum of intestinal and extra-intestinal endoparasites, reflecting their dual roles as apex predators and scavengers. Wolves and domestic dogs share a wide range parasite species, which is a reflection of their close genetic and taxonomic relationship (e.g., *Trichuris vulpis*, *Crenosoma vulpis, Angiostrongylus vasorum*), especially in areas where they have overlapping habitats. This ecological interface facilitates cross-species transmission, contributing to the maintenance and spread of parasitic populations. Monitoring these infections in wolves is therefore important not only for understanding wildlife health and for conservation of this endangered species, but also for assessing potential risks to domestic animals and public health, especially regarding zoonotic parasites (e.g., *Toxocara canis,* family* Ancylostomatidae *[hookworms], *Capillaria aerophila, Echinococcus granulosus*). Among the latter, the *Trichinella* spp. reservoir is maintained in nature by a wide range of reservoir hosts, including carnivores. *Trichinella britovi* is the most commonly detected species in European wolves, whereas infection with *Trichinella spiralis* and *Trichinella pseudospiralis* has been occasionally reported in Italy [18, 19]. Monitoring the presence of *Trichinella* spp. is essential, as they exhibit varying pathogenic potential and may pose different levels of risk to human health.

The diffusion and circulation of arthropod vectors and parasitic agents, including those of zoonotic relevance, can be favored by several factors, such as climate change, anthropogenic landscape modifications that reduce natural habitats and increased interactions between domestic animals and wildlife. The occurrence of wolf–dog hybrids in central Italy [20] provides concrete evidence of such interactions. In this context, the concept of One Health comes into play: wildlife parasite surveillance is essential not only for safeguarding animal health and ecosystem management but also for preventing disease transmission to domestic animals and humans, especially in areas where humans, dogs and wolves live in proximity.

Therefore, the present study aimed at monitoring parasitic infections in wolves from central Italy, particularly those of zoonotic importance.

## Methods

### Animals and sampling

Data from 169 wolf carcasses submitted and examined as part of routine activities at the Istituto Zooprofilattico Sperimentale dell’Abruzzo e del Molise “G. Caporale” (IZSAM), Teramo, Italy were retrospectively analyzed. Specifically, carcasses were recovered from the Abruzzo region or near the regional border area between 2018 and 2024 by the local health authority and submitted to the IZSAM for investigations into the cause of death and wildlife surveillance programs. Samples were collected from animals found dead, primarily as a result of road traffic accidents. During dissection, a fecal sample from the rectal ampulla and a sample from the muscle of the forelimb were collected from each individual, stored at  ± 2–4 °C and examined within 24–48 h of delivery to the laboratory. Spleen samples were collected and stored at ≤  − 20 °C until molecular analysis. For each animal, sex and estimated age were recorded, as well as the geographical coordinates of the carcass location. The coordinates, expressed in the World Geodetic System 1984 (WGS84) reference system (EPSG:4326 geodetic coordinate system), were mapped using ArcMap 10.8.1 GIS software (Environmental Systems Research Institute, Inc. [ESRI], Redlands, CA, USA). The Corine Land Cover (CLC) classification, which identifies different types of land cover and is standardized at the European level, was used to categorize the distribution of positive and negative samples according to level-2 detail within the level-1 macro-categories: artificial surfaces (1), agricultural areas (2), forest and semi-natural areas (3), wetlands (4) and water bodies (5) (https://land.copernicus.eu/en/products/corine-land-cover/clc2018).

Wolves were classified into three age classes: juveniles (< 12 months), yearlings/sub-adults (12 to 24 months) and adults (> 24 months) [21, 22].

### Laboratory analysis

#### *Leishmania* spp.

A 1.5-cm^3^ portion of spleen was mixed with 10 ml of phosphate buffered saline, homogenized and centrifuged at 16,000 *g* for 10 min. DNA was extracted from the sediment using the standardized lysis and purification protocol of the Maxwell RSC® Instrument Genomic Kit (Promega, Madison, WI, USA). DNA was analyzed by real-time PCR (qPCR) [23] to detect *Leishmania* spp.

#### *Trichinella* spp.

Samples (1 to 15 g) of tissue from the forelimb muscle were analyzed for the presence of *Trichinella* spp. larvae using an artificial digestion/magnetic stirrer method for pooled sample digestion in accordance with the UNI EN ISO 18743:2015/Amd.1:2023 standard and Commission Implementing Regulation (EU) 2015/1375 (European Commission, 2015: https://eur-lex.europa.eu/eli/reg_impl/2015/1375/oj/eng). Following digestion, the larvae were isolated using a stereomicroscope. Samples were considered positive if ≥ 1 larvae were found. All positive samples were sent to the EU reference laboratory (Istituto Superiore di Sanità, Rome) for species identification by a multiplex PCR [24].

### Copromicroscopic examinations

Each fecal sample was analyzed using the flotation method and the Baermann technique.

For the flotation method, approximately 7 g of feces was thoroughly mixed with water to form a homogeneous suspension. The mixture was then filtered through a double layer of cotton gauze to remove large debris, transferred to a Falcon tube and centrifuged at 1000 rpm for 5 min. Following centrifugation, the supernatant was discarded, and a zinc sulfate solution with a specific gravity of 1300 was added to the sediment. The sample was centrifuged again, then more solution was added to the top of the tube to form a meniscus, after which a coverslip was placed at the top of the tube. After 3–5 min, the coverslip was transferred to a microscope slide and examined under a microscope [25, 26].

The Baermann test was performed by placing approximately 4 g of fecal material inside a double layer of cheesecloth, which was then tied to form a pouch. The fecal pouch was placed in a funnel, and the funnel was clamped shut and filled with water at room temperature. After 18–24 h, 10 ml of fluid was collected into a tube containing three drops of 10% Lugol’s iodine solution and centrifuged at 2000 rpm for 5 min. The sediment at the bottom of the tube was transferred to a slide and examined under a microscope [26, 27]. Parasitic elements were identified using morphological and morphometric keys [26, 27].

## Results

### Wolf population, cause of death and localization of carcasses

Of the 169 wolves included in the study, 89 (52.66%) were males, and 80 (47.3%) were females. In terms of age, 131 wolves (77.5%) were adults, 32 (18.93%) were juveniles and six (3.5%) were subadults/yearlings. The majority of the wolf carcasses (97.6%; 165/169) were recovered in the Abruzzo region; the remaining four (2.4%) carcasses were recovered in the nearby regions of Lazio and Molise.

**Fig. 1:**
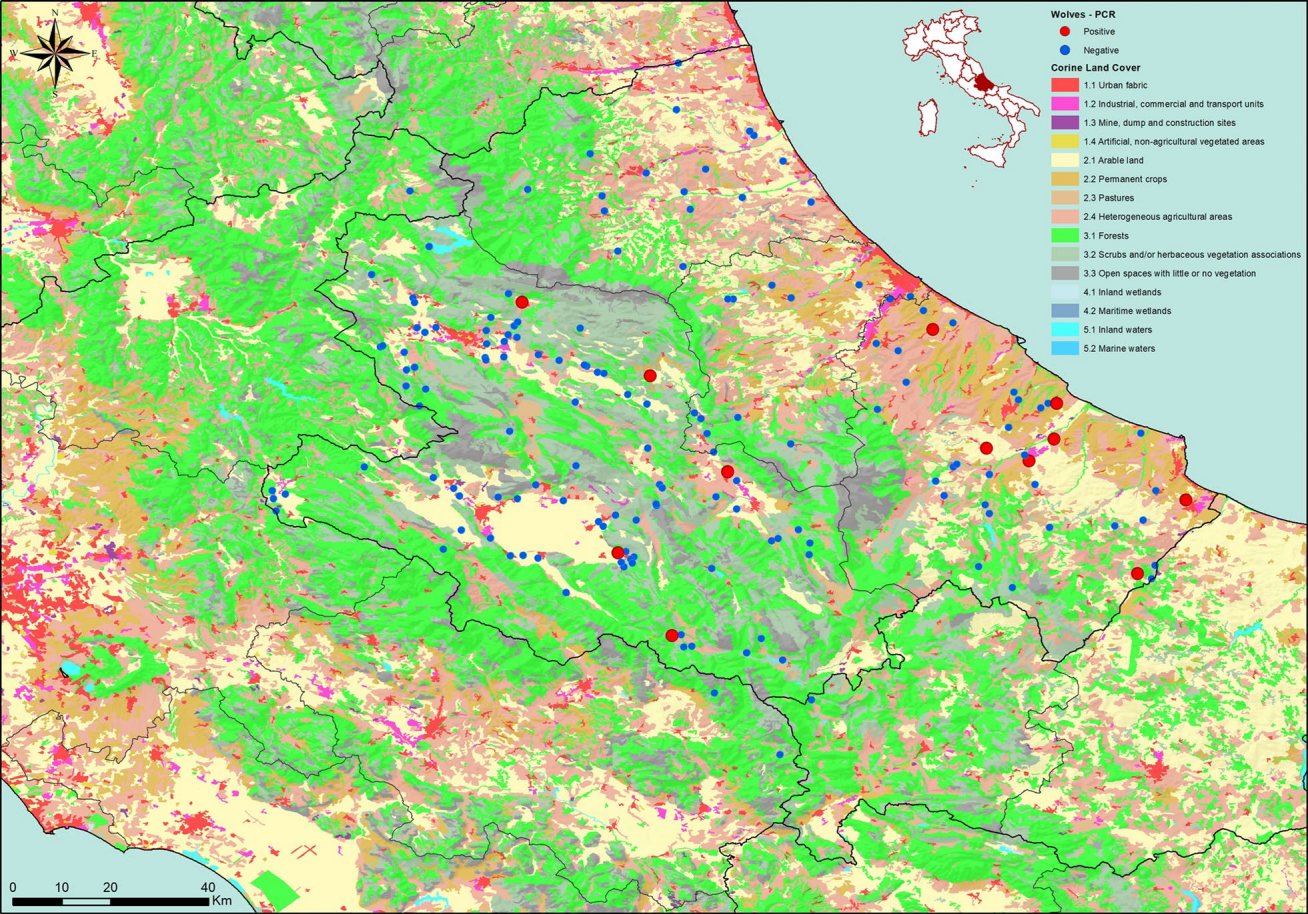
Geographical distribution of wolf carcasses recovered in the Abruzzo region (CLC map). The map is based on level 2 CLC classification within the level 1 macro-categories: artificial surfaces (1), agricultural areas (2) and forest and semi-natural areas (3). Each point represents a single wolf carcass, with blue dots indicating individuals that tested negative for *Leishmania* spp.*,* and red dots indicating positive cases. Wolves found near the border of the Abruzzo region were considered residents of protected areas within the region. CLC, Corine Land Cover

Post-mortem examination revealed that 147 (87%) wolves died from a traumatic cause (i.e. road killing), four (2.3%) died from infectious/parasitic diseases and 12 (7.1%) died from exposure to chemicals (e.g. poisoning) The cause of death remained undetermined for six (3.5%) animals. Twelve (7%) wolf carcasses were found on artificial surfaces, 116 (69%) were found in agricultural areas and 41 (24%) were found in forest and semi-natural areas (Table 1). The detailed geographic distribution of carcasses is shown in Fig. 1.

**Table 1:** Distribution of wolves by macro-categories and level 2 Corine Land Cover database codes

Macro-categories (level 1)	Level 2 CLC code and description	Negative, *n* (%)	Positive, *n* (%)	Total, *N* (%)
1—Artificial surfaces	1.1—Urban fabric	10 (6)	0 (0)	10 (6)
	1.2—Industrial, commercial and transport units	2 (1.2)	0 (0)	2 (1.2)
2—Agricultural areas	2.1—Arable land	36 (21.3)	5 (3)	41 (24.2)
	2.2—Permanent crops	12 (7.1)	1 (0.6)	13 (7.7)
	2.3—Pastures	2 (1.2)	0 (0)	2 (1.2)
	2.4—Heterogeneous agricultural areas	54 (32)	6 (3.5)	60 (35.5)
3—Forest and semi-natural areas	3.1—Forests	19 (11.2)	0 (0)	19 (11.2)
	3.2—Scrubs and/or herbaceous vegetation associations	17 (10)	0 (0)	17 (10)
	3.3—Open spaces with little or no vegetation	5 (3)	0 (0)	5 (3)
	Total no. (%)	157 (93)	12 (7.1)	169 (100)

*CLC* Corine Land Cover classification, *N (%)* total number (percentage) of samples within a specific category, *n (%)* number (percentage) of positive or negative samples within a specific category

### *Leishmania* spp., *Trichinella* spp. and copromicroscopic examinations

Of the 169 wolf carcasses analyzed, 135 (80%) tested positive for at least one parasite, including zoonotic parasites.

*Leishmania* spp. were tested for in all wolves, and 12 (7.1%) spleen samples were positive. Among the positive wolves, three had skin lesions consistent with *L. infantum* infection, such as dermatitis, alopecia, or crusty lesions (Table 2). All *Leishmania* spp.—positive wolves originated from agricultural areas, including arable lands, permanent crops, pastures and heterogeneous agricultural regions (Table 1).

**Table 2:** Characteristics of wolves found to be positive for *Leishmania* spp.

Cases	Sex	Estimated age^a^	BCS^b^	Place of recovery (province)	Lesions related to *Leishmania* spp. infection	Cause of death
1	M	Adult	3/5	AQ	–	Vehicle-related trauma
2	F	Adult	3/5	CH	–	Gunshot wound
3	F	Adult	2/5	CH	Alopecic and crusted areas on the head, thorax, distal limbs and sacral region	Vehicle-related trauma
4	F	Adult	3/5	AQ	–	Vehicle-related trauma
5	M	Adult	3/5	AQ	–	Gunshot wound
6	F	Adult	3/5	CH	–	Vehicle-related trauma
7	M	Adult	2/5	CH	–	Vehicle-related trauma
8	F	Adult	3/5	AQ	–	Vehicle-related trauma
9	M	Adult	3/5	CH	–	Vehicle-related trauma
10	F	Adult	3/5	CH	–	Vehicle-related trauma
11	M	Adult	3/5	CH	Alopecic areas on the neck, thorax, rump and distal limbs, associated with crusts	Vehicle-related trauma
12	M	Juvenile	2/5	AQ	Crusty dermatitis associated with diffuse alopecia on the whole body	Vehicle-related trauma

*AQ* L'Aquila,*BCS* Body condition score,* CH* Chieti,* F* female,* M* male

^a^Individuals were classified using methods previously validated for gray wolves [22]

^b^In the absence of a recognized and standardized BCS scale for the wolf, the canine scale was adopted, i.e., 1 = emaciated; 2 = thin; 3 = ideal; 4 = overweight; 5 = obese [28]

*Trichinell*a spp. detection was performed on 150 striated muscle samples harvested from wolves during wildlife surveillance programs. Of the 43 (28.66%) wolves that tested positive for *Trichinella* spp., 41 (95.3%) were infected with *T. britovi* as determined by multiplex-PCR (Table 3). Molecular identification of two samples was unsuccessful, likely due to the condition of the original sample. Fecal samples were collected from 147 of the 169 wolves; the rectal ampulla was found to be empty at necropsy in the remaining samples. Of these fecal samples from 147 wolves, 121 (82.3%) samples tested positive for at least one parasitic infection. Infection with nematodes belonging to family* Ancylostomatidae* was the most frequently detected infection (62%; 91/147), followed by infection by *T. vulpis* (32%; 47/147). Other parasitic infections detected by microscopic examination are shown in Table 3.

**Table 3:** Number (percentage) of wolves infected with intestinal and extra-intestinal parasites

Detection method	*N*	*n* (%)
*Flotation technique*		
Family* Ancylostomatidae* (hookworms)	147	91 (62)
*Toxocara canis*	147	3 (2)
Coccidian oocysts	147	7 (4.8)
*Trichuris vulpis*	147	47 (32)
*Capillaria* spp.	147	30 (20.4)
Class Cestoda	147	13 (8.8)
*Baermann test*		
* Angiostrongylus vasorum*	147	11 (7.5)
* Crenosoma vulpis*	147	4 (2.7)
Nematodes larvae^a^	147	5 (3.4)
*Molecular analyses*		
* Trichinella* spp.	150	43 (28.6)
* Trichinella britovi*	43	41 (95.3)
* Leishmania* spp.	169	12 (7.1)

^a^Identification not performed

*N* Total number of samples within a specific category, *n (%)* number (percentage) of positive or negative samples within a specific category

Of the 121 positive fecal samples, 51 (42.1%) had a single infection, and 70 (58%) were infected by more than one parasite. The most common co-infections were those of family Ancylostomatidae + *T. vulpis* (22/121, 18.2%), and family* Ancylostomatidae* + *Capillaria* spp. (15/121, 12.4%) (Table 4).

**Table 4:** Parasite species involved in mixed infections identified in wolf fecal samples (flotation and Baermann techniques)

Number of parasite species	Mixed infections	Total number of wolves = 121, *n* (%)
2	Family* Ancylostomatidae*, *Capillaria* spp.	15 (12.4)
2	Family* Ancylostomatidae*, *Crenosoma vulpis*	1 (0.8)
2	Family* Ancylostomatidae*, *Trichuris vulpis*	22 (18.2)
2	Family* Ancylostomatidae*, Class Cestoda	3 (2.5)
2	Family* Ancylostomatidae*, Coccidian oocysts	1 (0.8)
2	*Angiostrongylus vasorum*, *Trichuris vulpis*	3 (2.5)
2	*Capillaria* spp.*, Angiostrongylus vasorum*	1 (0.8)
2	*Capillaria* spp.*,* Class Cestoda	1 (0.8)
2	*Capillaria* spp., Coccidian oocysts	1 (0.8)
2	Coccidian oocysts, *Trichuris vulpis*	1 (0.8)
2	*Crenosoma vulpis*, nematodes larvae^a^	1 (0.8)
3	*Crenosoma vulpis, Trichuris vulpis*	1 (0.8)
2	*Trichuris vulpis*; nematodes larvae^a^	1 (0.8)
3	Family* Ancylostomatidae*, *Angiostrongylus vasorum*, *Capillaria* spp.	3 (2.5)
3	Family* Ancylostomatidae*, *Angiostrongylus vasorum*, *Trichuris vulpis*	2 (1.6)
3	Family Ancylostomatidae*, Capillaria* spp., Coccidian oocysts	1 (0.8)
3	Family* Ancylostomatidae*, *Capillaria* spp., nematodes larvae^a^	1 (0.8)
3	Family* Ancylostomatidae, Capillaria* spp., *Toxocara canis*	1 (0.8)
3	Family* Ancylostomatidae*, *Capillaria* spp., *Trichuris vulpis*	2 (1.6)
3	Family* Ancylostomatidae*, *Crenosoma vulpis*, *Trichuris vulpis*	1 (0.8)
3	Family* Ancylostomatidae*, Class Cestoda, *Trichuris vulpis*	2 (1.6)
3	Family* Ancylostomatidae*, Coccidian oocysts, *Trichuris vulpis*	1 (0.8)
3	Family* Ancylostomatidae*, nematodes larvae^a^, *Trichuris vulpis*	2 (1.6)
4	Family* Ancylostomatidae*, *Angiostrongylus vasorum*, *Trichuris vulpis*, *Toxocara canis*	1 (0.8)
4	Family* Ancylostomatidae*, Class Cestoda, *Trichuris vulpis*, coccidian oocysts	1 (0.8)
*Total number of wolves with mixed infections*		70 (58)

*N* Total number of wolves, *n(%)* number (percentage) of positive or negative samples within a specific category

^a^Identification not performed

## Discussion

The findings of this study demonstrate a high prevalence of various parasitic species in the wolf population of central Italy, including several with zoonotic potential, highlighting the relevance of wolves as sentinels for parasite circulation at the wildlife–human interface.

*Leishmania infantum* is endemic throughout the Mediterranean basin. Its prevalence in the canine population varies significantly across European regions (from 2% to 30%), with the highest prevalences estimated in France, Italy and Spain, and the lowest recorded in Slovenia [29].

To the best of the authors’ knowledge, this is the first epidemiological estimate of *Leishmania* spp. infection in wolves from central Italy, with the results of the analyses showing a prevalence of 7.1% (12/169 carcasses). Prior data from Italy were based on a limited sample size. In northeastern Italy (Emilia-Romagna region), molecular testing of earlobe tissue detected *L. infantum* in three of 18 wolves (16.7%), while spleen samples from the same animals tested negative [11]. In contrast, in northwestern Italy (Piemonte region), PCR analysis performed on splenic tissue identified *L. infantum* DNA in seven of 33 wolves (21.2%) [30]. Although species identification by genotyping was not performed in the present study, epidemiological data from the literature suggest that the wolves testing positive for *Leishmania* spp. infection were most likely infected with *L. infantum*.

Moreover, to date, this cohort (*n* = 169) constitutes, to the best of the authors’ knowledge, the largest European wolf sample investigated for parasitic infections. However, direct comparisons with most studies are limited by the variability in diagnostic methods applied (serology vs. molecular assays) and in the tissues analyzed (e.g. lymph nodes, earlobe skin, blood, spleen) [17, 31]. A similar study conducted in Asturias (northern Spain), an area considered to be non-endemic for parasites, examined 102 splenic tissues from wolves using molecular methods and found a much higher prevalence (33%) of parasitic infections than in the present study (7.1%) [32]. This discrepancy may reflect variation in test sensitivity and tissue tropism, as well as genuine geographic heterogeneity in parasite circulation, as documented in recent years among humans and hares in central Spain [33–35]. These findings highlight the need for standardized sampling and diagnostic protocols when comparing prevalence data across studies and regions.

Although available data are limited, *L. infantum* usually causes subclinical or paucisymptomatic infections in wolves. A wolf with fatal visceral leishmaniosis, showing clinicopathological changes similar to those observed in canine disease, including dermatitis, alopecia, cachexia and hepatosplenomegaly, was reported in a single case report [36]. In the present study, two of the three *Leishmania* spp.-positive wolves with skin lesions were co-infected with *Sarcoptes scabiei*, so it was not possible to attribute the lesions with absolute certainty to leishmaniosis rather than to mite infestation. Moreover, the cause of death in all 12 *L. infantum* spp.-positive wolves was traumatic. Overall, *L. infantum* infection does not appear to pose a lethal threat to wolf populations, although indirect effects, such as impaired body condition, predisposition to secondary diseases or reduced hunting performance, cannot be excluded. Despite the detection of *L. infantum* across multiple studies, the epidemiological role of the wolf in disease circulation remains uncertain. Studies are needed to understand the role of wildlife in the transmission of leishmaniosis and to inform comprehensive control strategies, particularly in regions where wildlife, domestic animals and humans coexist closely [17]. Notably, most wolves included in the study, and all wolves that tested positive for *Leishmania* spp., were detected in agricultural areas, where interactions with domestic dogs are more likely than in forest and semi-natural areas. This finding highlights the need for management plans to reduce contacts and conflicts with humans and domestic animals [37]. Additionally, half of the positive animals (and several negative animals) were detected in the coastal area, supporting ongoing range expansion beyond the mountain belt.

All parasites detected in wolves by copromicroscopic examination in the present study also infect domestic dogs. Consequently, the proximity of wolves to anthropized areas, as well as the presence of poorly managed or free-roaming dogs with unrestricted access to natural habitats, likely facilitates bidirectional transmission of these parasites, with possible public health implications and a potential impact on species conservation. In the present study, we recorded the highest prevalence (62%) of infection with members of the family Ancylostomatidae reported to date, exceeding rates reported in wolves from northern and central Italy and Poland, Latvia, Greece, Spain and Portugal, where hookworms were also the most prevalent nematodes, albeit with variable prevalences (up to 40%) [37–44]. Although species-level identification was not undertaken in the present survey, previous investigations in wolf populations have reported infections with *A. caninum* and *Uncinaria stenocephala*, both belonging to the Family Ancylostomatidae [37–43]. Their widespread distribution in the wolf population likely reflects multiple transmission routes, including ingestion of paratenic hosts or ingestion or percutaneous penetration of third-stage larvae (L3) from the environment. Lactogenic vertical transmission has also been demonstrated for For *A. caninum* [27, 45]. While the zoonotic significance of nematodes belonging to Family Ancylostomatidae currently remains incompletely understood, several other parasites detected in wolves can cause severe, potentially life-threatening diseases in humans [46].

The prevalence of ascarid infections (2%) recorded in the present study is similar to that reported in another recent study from central Italy (1.5% in Tuscany) [44], but lower than those reported in previous studies in Italy and Europe (5–10%) [5, 40, 43]. Although wolves can also harbor *Toxascaris leonina* [40, 43], we only identified *T. canis* in our study. Special attention should be paid to this pathogen, given its high prevalence in canine populations and zoonotic potential.

Eggs of *T. canis* can be misdiagnosed with those of *Baylisascaris procyonis*, a nematode of serious zoonotic concern that can cause severe and often fatal neural larva migrans in humans [46–48]. Continuous surveillance is therefore strongly recommended because *B. procyonis* has recently been detected in wolf feces in France. Although wolves are not currently considered to be definitive hosts of this parasite, the parasite is expanding across Europe. Its natural host, the raccoon (*Procyon lotor*), is an invasive alien species in Italy, where an official eradication program is currently in place; notably, a high percentage (33%) of raccoons captured in the Tuscany region (central Italy) tested positive for *B. procyonis* [49–52].

The high proportion of *Trichinella* spp.-positive wolves (28.7%) observed in our study confirms the stable circulation of this parasite in central Italy and is consistent with previous estimates from central and southern Italy (21.9–30%) [18, 53, 54]. The higher prevalence in wolves compared with species at lower trophic levels (e.g. wild boars, foxes) is consistent with their ecological role [53–56]. As apex predators and opportunistic scavengers, wolves are more likely to ingest infected tissue from diverse prey species, and their longer lifespan (vs. foxes) may allow greater accumulation of *Trichinella* larvae over time [57]. Although *Trichinella* spp. infection in wolves does not itself pose a direct risk to humans, the high prevalence suggests active circulation of the parasite in hunting areas and, therefore, a potential risk of human trichinellosis via consumption of raw or undercooked meat from infected game. While only *T. britovi* was detected in the present study, continued surveillance with species-level identification remains imperative, particularly in light of a recent report of *T. spiralis* infection in a wolf from central Italy (Lazio region) [19], a species associated with higher pathogenicity in humans*.*

The prevalence of *A. vasorum* (7.5%) in this study is higher than that reported recently in dogs from Abruzzo (1.5%) and Molise (0.5%), but lower than that reported in the Umbria region (10%) [58]. Previous studies in central Italy (Abruzzo, Lazio, Toscana) reported a wide variation in the prevalence of *A. vasorum* in wolves, with prevalence ranging from 1.5 to 66.7% [44, 59–61]. The highest detection rate of *A. vasorum* was observed in a smaller subset of wolves examined using a combination of diagnostic methods, namely the Baermann test and direct lung-tissue analysis; the latter can identify infections even when fecal larval shedding is absent [61]. Prevalence data on *A. vasorum* infection in wolves from the Mediterranean Basin are limited. Higher prevalences (19–22%) were reported in Iberian wolves (*Canis lupus signatus*) using direct examination of cardiac chambers and blood vessels combined with the cup sedimentation technique (immersion of the lungs in water, subsequent collection of the sediment and microscopic examination) and/or enzymatic digestion in pepsin and chlorhydric acid [62, 63]. Combinations of these techniques may be more sensitive than the Baermann method alone and could account for the higher prevalence observed, as previously discussed in Italian wolves. Accordingly, none of the fecal samples from Iberian wolves collected from the environment (*n* = 11) and examined using the Baermann technique in a Portuguese study tested positive for *A. vasorum* [64]. Another study of 400 individual fecal samples from Croatian wild wolves detected a prevalence of *A. vasorum* of 3.1%; however, the diagnostic technique employed (i.e. the sodium acetate–acetic acid–formalin [SAF] technique) is not considered the gold standard for diagnosing angiostrongylosis, and therefore a possible underestimation of positive cases cannot be excluded [65].

While direct comparisons between these studies are limited by differences in protocols, these findings indicate that *A. vasorum* is consistently present in wolf populations in central Italy, suggesting that this wild canid could contribute to the parasite's environmental maintenance. However, to date, it remains unclear whether the wolf represents an ancestral host of *A. vasorum* or whether the infections detected are the result of more recent spillover from foxes (the natural host) and dogs. Although *A. vasorum* is not zoonotic, its importance lies in its spread in the canine population and in the severe, sometimes fatal disease it can cause in dogs (respiratory distress, coagulopathies, neurologic signs). Mapping its occurrence in wolves is therefore relevant to understanding transmission among wild and domestic canids that share the same habitats. In fact, although the pathogenic potential of *A. vasorum* in dogs is well established, data on the disease in wolves remain extremely limited. Nevertheless, it is highly plausible that the parasite can induce clinical manifestations in wolves similar to those observed in domestic dogs, potentially resulting in severe and even fatal disease. Therefore, surveillance of angiostrongylosis is of paramount importance not only in domestic animals, for which effective prophylactic and therapeutic measures are available, but also—perhaps most critically—in wolf populations. In this species, the spread of the infection could represent an additional threat, potentially affecting population health and long-term conservation.

The prevalence of *C. vulpis* detected in the present study was 2.7%, which is within the range reported in the Tuscany region, Italy (1.5–13.15%) [5, 44]. *Crenosoma vulpis* is a respiratory nematode of lesser importance in veterinary medicine compared to *A. vasorum*. It typically causes non-fatal chronic bronchitis characterized by a persistent cough, and it is less prevalent in the canine population because red foxes are its natural hosts. However, the diagnosis is performed using the Baermann technique, similar to *A. vasorum*; therefore, detailed knowledge of morphological and morphometric features of the larvae is crucial for accurate species identification and appropriate treatment strategies.

Available data on *C. vulpis* in European wolves are lacking. In the same aforementioned studies on Iberian wolves, prevalence rates from 7% to 9% were reported, which are higher than those observed in the present study. As for angiostrongylosis, this difference may be explained by differences in the sensitivity of the diagnostic techniques used [62, 63]. The prevalence detected by the Baermann technique in fecal samples from Iberian wolves collected in an area of Portugal is similar to that in Spain (9%) [64]; however, in that study, the sample size was very small (11 wolves). The observed prevalence of *C. vulpis* in Croatian wolves [65] is similar (i.e. 4.6%) to that reported in the present study. However, differences in the techniques used, the number of samples, and the target populations across these studies prevent a direct comparison.

Although larvae of *A. vasorum* and *C. vulpis* can remain alive and viable in the environment under different humidity and temperature conditions [66–68], it cannot be ruled out that performing the Baermann technique on fecal samples collected from carcasses that were not always fresh may have influenced the results, potentially leading to an underestimation of the prevalence of *A. vasorum* and *C. vulpis* in the studied population. However, this hypothesis appears less likely, considering that in the life-cycle of these parasites, first-stage larvae are shed into the environment, where they can survive for variable periods; consequently, their detection in fecal samples is not strictly dependent on immediate sample freshness. Therefore, while some degree of underestimation cannot be ruled out—also because, as discussed, the Baermann technique may be less sensitive than the direct detection of parasites in lung tissues—the prevalence values obtained in this study are nonetheless likely to be broadly representative of the true infection status of the population examined.

Several *Capillaria* species can infect both domestic and wild mammals. The most important are *C. aerophila* and *Capillaria boehmi*, two respiratory nematodes commonly found in red foxes, which can also cause disease in pets. The presence of these two species in wolves is poorly documented. As species-level identification was not performed in the present study, it was impossible to determine whether the findings represent actual infection or pseudoparasitism [40, 41, 69]. Nevertheless, the high prevalence observed in the present (20.4%) and past Italian studies (55.2–81.8%) [5, 44] warrants attention. In the Mediterranean basin, specifically in Spain, adults of *C. aerophila* were detected in 3.5–5% of examined wolves using direct lung inspection, cup sedimentation, and enzymatic digestion of lungs [62, 63]. In a Portuguese study, all 11 environmental fecal samples attributed to the Iberian wolf were negative for *Capillaria* spp. [64], while a prevalence of 16% was recorded in fecal samples from wild Croatian wolves [65]. Further studies are needed to clarify which species are truly capable of establishing infection and causing disease in wolves, including consideration of the zoonotic potential of particular species, such as *C. aerophila*.

The detection of helminth eggs belonging to the Class Cestoda is particularly noteworthy, as copromicroscopy alone does not allow species-level identification. *Echinococcus granulosus* sensu lato infection has a significant impact on human health, causing a worldwide-distributed, potentially fatal disease. Its eggs are indistinguishable from those of other taeniids, and species-level identification requires molecular methods [70]. Infections with helminths belonging to Class Cestoda occur via predation or ingestion of infected intermediate hosts, ranging from small rodents to large wild or domestic herbivores. Although wolves are apex predators, available evidence suggests they play a limited role in the life-cycle of *E. granulosus* [48].

The prevalence of cestode-positive samples (8.8%) in this study was substantially lower than that reported in central and northern Italy over the past decade, where cestodes have been detected in up to 30% of wolves [40, 44, 71–74]. Differences in diagnostic methodology and the possibility of intermittent egg shedding should be considered when interpreting these contrasting findings. Conventional copromicroscopy may underestimate positivity compared to molecular methods [73, 74]. Notably, in a recent study of wolves from Abruzzo, *E. granulosus* DNA was detected in one of 20 fecal samples (5%) [60]. Therefore, further molecular studies are needed to clarify the circulation of cestodes, particularly of *E. granulosus,* in wolf populations and to update estimates in central Italy [73, 74]. Moreover, a limitation of the study is that adult cestode specimens were not collected during necropsy, although their recovery represents an accurate method for cestode identification.

## Conclusions

The high prevalence of parasitic infections detected in wolves in Italy highlights the critical importance of wildlife parasite monitoring, not only to ensure the conservation and health of wolf populations, but also to preserve ecosystem integrity and to better understand the ecology and biology of parasites and host–pathogen dynamics. Within the One Health framework, these efforts become even more essential, recognizing the interconnected health of wildlife, livestock, humans and the environment. Finally, given the increasing anthropogenic pressures driving wild animals into urban and peri‑urban areas, intensified surveillance is crucial to the control of both established and emerging zoonotic risks at the human–wildlife interface.

## Data Availability

The dataset supporting the conclusions of this article is included within the article.

## Abbreviations

CLC: Corine Land Cover classification
IZSAM: Istituto Zooprofilattico Sperimentale dell’Abruzzo e del Molise “G. Caporale”

## References

[1] Lucchini V, Galov A, Randi E. Evidence of genetic distinction and long-term population decline in wolves (*Canis lupus*) in the Italian Apennines. Mol Ecol. 2004;13:523–36. 14871358 10.1046/j.1365-294x.2004.02077.x

[2] Breitenmoser U. Large predators in the Alps: the fall and rise of man’s competitors. Biol Conserv. 1998;83:279–89.

[3] Ciancio O, Corona P, Lamonaca A, Portoghesi L, Travaglini D. Conversion of clearcut beech coppices into high forests with continuous cover: a case study in central Italy. For Ecol Manag. 2006;224:235–40.

[4] Hassell JM, Begon M, Ward MJ, Fèvre EM. Urbanization and disease emergence: dynamics at the wildlife-livestock-human interface. Trends Ecol Evol. 2017;32:55–67. 28029378 10.1016/j.tree.2016.09.012 PMC5214842

[5] Macchioni F, Coppola F, Furzi F, Gabrielli S, Baldanti S, Boni CB, et al. Taeniid cestodes in a wolf pack living in a highly anthropic hilly agro-ecosystem. Parasite. 2021;28:10. 33544075 10.1051/parasite/2021008 PMC7863970

[6] Millán J, Ferroglio E, Solano-Gallego L. Role of wildlife in the epidemiology of *Leishmania infantum* infection in Europe. Parasitol Res. 2014;113:2005–14. 24804923 10.1007/s00436-014-3929-2

[7] Berriatua E, Maia C, Conceição C, Özbel Y, Töz S, Baneth G, et al. Leishmaniases in the European Union and neighboring countries. Emerg Infect Dis. 2021;27:1723–7. 34013857 10.3201/eid2706.210239 PMC8153892

[8] Carvalho BM, Maia C, Courtenay O, Llabrés-Brustenga A, Lotto Batista M, Moirano G, et al. A climatic suitability indicator to support *Leishmania infantum* surveillance in Europe: a modelling study. Lancet Reg Health Eur. 2024;43:100971. 39040529 10.1016/j.lanepe.2024.100971 PMC11261136

[9] Mendoza-Roldan J, Benelli G, Panarese R, Iatta R, Furlanello T, Beugnet F, et al. *Leishmania infantum* and *Dirofilaria immitis* infections in Italy, 2009–2019: changing distribution patterns. Parasit Vectors. 2020;13:193. 32293524 10.1186/s13071-020-04063-9 PMC7161282

[10] Castelli G, Bruno F, Caputo V, Fiorella S, Sammarco I, Lupo T, et al. Genetic tools discriminate strains of *Leishmania infantum* isolated from humans and dogs in Sicily, Italy. PLoS Negl Trop Dis. 2020;14:e0008465. 32706789 10.1371/journal.pntd.0008465 PMC7406075

[11] Taddei R, Bregoli A, Galletti G, Carra E, Fiorentini L, Fontana MC, et al. Wildlife hosts of* Leishmania infantum* in a re-emerging focus of human leishmaniasis, in Emilia-Romagna, Northeast Italy. Pathogens. 2022;11:1308. 10.3390/pathogens11111308. 10.3390/pathogens11111308 PMC9697138 36365059

[12] Barroso P, Zanet S, Ferroglio E. Meteorological, demographic, and environmental risk factors for *Leishmania infantum* in wildlife. Res Vet Sci. 2024;173:105288. 38718544 10.1016/j.rvsc.2024.105288

[13] Mancianti F, Mignone W, Galastri F. Serologic survey for leishmaniasis in free-living red foxes (*Vulpes vulpes*) in Italy. J Wildl Dis. 1994;30:454–6. 7933296 10.7589/0090-3558-30.3.454

[14] Dipineto L, Manna L, Baiano A, Gala M, Fioretti A, Gravino AE, et al. Presence of *Leishmania infantum* in red foxes (*Vulpes vulpes*) in southern Italy. J Wildl Dis. 2007;43:518–20. 17699092 10.7589/0090-3558-43.3.518

[15] Verin R, Poli A, Ariti G, Nardoni S, Fanucchi MB, Mancianti F. Detection of *Leishmania infantum* DNA in tissues of free-ranging red foxes (*Vulpes vulpes*) in Central Italy. Eur J Wildl Res. 2010;56:689–92.

[16] Piantedosi D, Veneziano V, Di Muccio T, Manzillo VF, Fiorentino E, Scalone A, et al. Epidemiological survey on *Leishmania* infection in red foxes (*Vulpes vulpes*) and hunting dogs sharing the same rural area in Southern Italy. Acta Parasitol. 2016;61:769–75. 27787204 10.1515/ap-2016-0106

[17] Cardoso L, Schallig H, Persichetti MF, Pennisi MG. New epidemiological aspects of animal leishmaniosis in Europe: the role of vertebrate hosts other than dogs. Pathogens. 2021;10:307. 10.3390/pathogens10030307. 10.3390/pathogens10030307 PMC8000700 33800782

[18] Ricchiuti L, Petrini A, Interisano M, Ruberto A, Salucci S, Marino L, et al. First report of *Trichinella pseudospiralis* in a wolf (*Canis lupus italicus*). Int J Parasitol Parasites Wildl. 2021;15:195–8. 34136345 10.1016/j.ijppaw.2021.05.002 PMC8182262

[19] Marucci G, Raso C, Borgogni E, Celani F, Tartarelli I, Cherchi S, et al. First report of *T. spiralis* in a wolf in Italy: an increasing health concern? Food Waterborne Parasitol. 2025;38:e00253. 39835170 10.1016/j.fawpar.2024.e00253 PMC11743876

[20] Meriggi A, Dagradi V, Dondina O, Perversi M, Milanesi P, Lombardini M, et al. Short-term responses of wolf feeding habits to changes of wild and domestic ungulate abundance in Northern Italy. Ethol Ecol Evol. 2015;27:389–411.

[21] Sgroi G, D’Alessio N, Vada R, Ferroglio E, Vicente J, Veneziano V. The contribution of citizen science in the surveillance of wildlife and related arthropods. Parasitology. 2023;150:1089–95. 37929599 10.1017/S0031182023001038 PMC10801373

[22] Gipson PS, Ballard WB, Nowak RM, Mech LD. Accuracy and precision of estimating age of gray wolves by tooth wear. J Wildl Manag. 2000;64:752–8.

[23] Lombardo G, Pennisi MG, Lupo T, Migliazzo A, Caprì A, Solano-Gallego L. Detection of *Leishmania infantum* DNA by real-time PCR in canine oral and conjunctival swabs and comparison with other diagnostic techniques. Vet Parasitol. 2012;184:10–7. 21906883 10.1016/j.vetpar.2011.08.010

[24] Istituto Superiore di Sanità. Identificazione A Livello Di Specie Di Larve Di Trichinella Mediante Multiplex-Pcr. 2024. https://www.iss.it/documents/20126/0/MI-02+%2810%29.pdf/45df1181-2b46-68a0-8ca8-f99c20e2b30f?t=1741690950834. Accessed 4 Dec 2025.

[25] Zajac AM, Johnson J, King SE. Evaluation of the importance of centrifugation as a component of zinc sulfate fecal flotation examinations. J Am Anim Hosp Assoc. 2002;38:221–4. 12022406 10.5326/0380221

[26] Zajac AM, Conboy GA, Little SE, Reichard MV. Veterinary clinical parasitology. New York: Wiley; 2021.

[27] Taylor AM, Coop LR, Wall LR. Veterinary parasitology. 4th ed. Milton: Blackwell Publishing; 2016.

[28] German AJ, Holden SL, Moxham GL, Holmes KL, Hackett RM, Rawlings JM. A simple, reliable tool for owners to assess the body condition of their dog or cat. J Nutr. 2006;136:2031s-s2033. 16772488 10.1093/jn/136.7.2031S

[29] Ferdes I, Medrouh B, Hakem A, Lafri I. Seroprevalence and risk factors of canine leishmaniasis in Mediterranean countries: a systematic review and meta-analysis (2000-2024). Res Vet Sci. 2025;194:105836. 40759080 10.1016/j.rvsc.2025.105836

[30] Battisti E, Zanet S, Khalili S, Trisciuoglio A, Hertel B, Ferroglio E. Molecular survey on vector-borne pathogens in alpine wild carnivorans. Front Vet Sci. 2020;7:1. 32039255 10.3389/fvets.2020.00001 PMC6989405

[31] Sastre N, Francino O, Ramírez O, Enseñat C, Sánchez A, Altet L. Detection of *Leishmania infantum* in captive wolves from Southwestern Europe. Vet Parasitol. 2008;158:117–20. 18823711 10.1016/j.vetpar.2008.08.008

[32] Oleaga A, Zanet S, Espí A, de Pegoraro Macedo MR, Gortázar C, Ferroglio E. *Leishmania* in wolves in northern Spain: a spreading zoonosis evidenced by wildlife sanitary surveillance. Vet Parasitol. 2018;255:26–31. 29773132 10.1016/j.vetpar.2018.03.015

[33] Molina R, Jiménez MI, Cruz I, Iriso A, Martín-Martín I, Sevillano O, et al. The hare (*Lepus granatensis*) as potential sylvatic reservoir of *Leishmania infantum* in Spain. Vet Parasitol. 2012;190:268–71. 22677135 10.1016/j.vetpar.2012.05.006

[34] Vilas F, Hervás JC, Sevilla S, Martínez A, Gavín MO, Bernal J, et al. Brote de Leishmaniasis en la zona suroeste de la Comunidad de Madrid: Medidas de investigación y control medioambiental. Prof Vet. 2012;17:6–15.

[35] Arce A, Estirado A, Ordobas M, Sevilla S, García N, Moratilla L, et al. Re-emergence of leishmaniasis in Spain: community outbreak in Madrid, Spain, 2009 to 2012. Euro Surveill. 2013;18:20546. 23929177 10.2807/1560-7917.es2013.18.30.20546

[36] Beck A, Beck R, Kusak J, Gudan A, Martinkovic F, Artukovic B, et al. A case of visceral leishmaniosis in a gray wolf (*Canis lupus*) from Croatia. J Wildl Dis. 2008;44:451–6. 18436678 10.7589/0090-3558-44.2.451

[37] Gervasi V, Aragno P, Salvatori V, Caniglia R, De Angelis D, Fabbri E, et al. Estimating distribution and abundance of wide-ranging species with integrated spatial models: opportunities revealed by the first wolf assessment in south-central Italy. Ecol Evol. 2024;14:e11285. 38746543 10.1002/ece3.11285 PMC11091487

[38] Bagrade G, Kirjusina M, Vismanis K, Ozoliņs J. Helminth parasites of the wolf *Canis lupus* from Latvia. J Helminthol. 2009;83:63–8. 19138449 10.1017/S0022149X08123860

[39] Diakou A, Karaiosif R, Petridou M, Iliopoulos Y, editors. Endoparasites of the wolf (*Canis lupus*) in central Greece. Poster No 113. EWDA 2014—11th European wildlife disease association conference, 25–29 August, 2014, Edinburgh.

[40] de Macedo MRP, Zanet S, Bruno S, Tolosano A, Marucco F, Rossi L, et al. Gastrointestinal helminths of wolves (*Canis lupus* Linnaeus, 1758) in Piedmont, north-western Italy. J Helminthol. 2019;94:e88. 31537202 10.1017/S0022149X19000841

[41] Molnar B, Ciucci P, Mastrantonio G, Betschart B. Correlates of parasites and pseudoparasites in wolves (*Canis lupus*) across continents: a comparison among Yellowstone (USA), Abruzzo (IT) and Mercantour (FR) national parks. Int J Parasitol Parasites Wildl. 2019;10:196–206. 31667082 10.1016/j.ijppaw.2019.09.002 PMC6812024

[42] Pereira AL, Mateus TL, Llaneza L, Vieira-Pinto MM, de Maira Carvalho LM. Gastrointestinal parasites in iberian wolf (*canis lupus signatus*) from the Iberian Peninsula. Parasitologia. 2023;3:15–32.

[43] Perrucci S, Maestrini M, Coppola F, Di Marco M, Rosso AD, Pacini MI, et al. Gray wolf (*Canis lupus italicus*) and red fox (*Vulpes vulpes*) parasite survey in anthropized and natural areas of central Italy. Vet Sci. 2023;10:108. 36851412 10.3390/vetsci10020108 PMC9963820

[44] Cafiero SA, Petroni L, Natucci L, Casale L, Raffaelli M, Baldacci D, et al. Parasite diversity in grey wolves (*Canis lupus*) from Tuscany, central Italy: a copromicroscopical investigation. Int J Parasitol Parasites Wildl. 2025;27:101092. 40606262 10.1016/j.ijppaw.2025.101092 PMC12213270

[45] Zajac M, Conboy AG. Veterinary clinical parasitology. 8th ed. Oxford: Willey; 2012.

[46] Morelli S, Diakou A, Di Cesare A, Colombo M, Traversa D. Canine and feline parasitology: analogies, differences, and relevance for human health. Clin Microbiol Rev. 2021;34:e0026620. 34378954 10.1128/CMR.00266-20 PMC8404700

[47] Lee AC, Schantz PM, Kazacos KR, Montgomery SP, Bowman DD. Epidemiologic and zoonotic aspects of ascarid infections in dogs and cats. Trends Parasitol. 2010;26:155–61. 20172762 10.1016/j.pt.2010.01.002

[48] Umhang G, Duchamp C, Boucher JM, Caillot C, Legras L, Demerson JM, et al. Gray wolves as sentinels for the presence of *Echinococcus* spp. and other gastrointestinal parasites in France. Int J Parasitol Parasites Wildl. 2023;22:101–7. 37780970 10.1016/j.ijppaw.2023.09.007 PMC10539616

[49] Morelli S, Diakou A, Colombo M, Di Cesare A, Barlaam A, Dimzas D, et al. Cat respiratory nematodes: current knowledge, novel data and warranted studies on clinical features, treatment and control. Pathogens. 2021;10:454. 10.3390/pathogens10040454. 10.3390/pathogens10040454 PMC8069686 33920104

[50] Gavin PJ, Kazacos KR, Shulman ST. Baylisascariasis. Clin Microbiol Rev. 2005;18:703–18. 16223954 10.1128/CMR.18.4.703-718.2005 PMC1265913

[51] Lombardo A, Brocherel G, Donnini C, Fichi G, Mariacher A, Diaconu EL, et al. First report of the zoonotic nematode *Baylisascaris procyonis* in non-native raccoons (*Procyon lotor*) from Italy. Parasit Vectors. 2022;15:24. 35022078 10.1186/s13071-021-05116-3 PMC8756652

[52] Heddergott M, Lippert S, Schliephake A, Gaede W, Schleimer A, Frantz AC. Spread of the zoonotic nematode *Baylisascaris procyonis* into a naive raccoon population. EcoHealth. 2023;20:263–72. 37971598 10.1007/s10393-023-01655-6 PMC10757695

[53] Badagliacca P, Di Sabatino D, Salucci S, Romeo G, Cipriani M, Sulli N, et al. The role of the wolf in endemic sylvatic *Trichinella britovi* infection in the Abruzzi region of Central Italy. Vet Parasitol. 2016;231:124–7. 27522469 10.1016/j.vetpar.2016.07.030

[54] Scarcelli S, Buono F, D’Alessio N, Rea S, Castaldo E, Pesce A, et al. *Trichinella* spp. in wolves (*Canis lupus*) and red foxes (*Vulpes vulpes*) of southern Italy. Res Vet Sci. 2024;179:105381. 39213743 10.1016/j.rvsc.2024.105381

[55] van der Giessen JW, Rombout Y, van der Veen A, Pozio E. Diagnosis and epidemiology of *Trichinella infections* in wildlife in The Netherlands. Parasite. 2001;8:S103–5. 11484327 10.1051/parasite/200108s2103

[56] Sgroi G, D’Alessio N, Marucci G, Pacifico L, Buono F, Deak G, et al. *Trichinella britovi* in wild boar meat from Italy, 2015–2021: a citizen science approach to surveillance. One Health. 2023;16:100480. 36632478 10.1016/j.onehlt.2022.100480 PMC9826805

[57] Martínez-Carrasco C, Moroni B, García-Garrigós A, Robetto S, Carella E, Zoppi S, et al. Wolf is back: a novel sensitive sentinel rejoins the *Trichinella* cycle in the Western Alps. Vet Sci. 2023;10:206. 10.3390/vetsci10030206. 10.3390/vetsci10030206 PMC10055899 36977245

[58] Traversa D, Morelli S, Di Cesare A, Astuti C, Barlaam A, Colombo M, et al. Current enzooticity of *Dirofilaria immitis* and *Angiostrongylus vasorum* in Central and Southern Italy. Animals. 2025;15:172. 39858171 10.3390/ani15020172 PMC11758344

[59] De Liberato C, Grifoni G, Lorenzetti R, Meoli R, Cocumelli C, Mastromattei A, et al. *Angiostrongylus vasorum* in wolves in Italy: prevalence and pathological findings. Parasit Vectors. 2017;10:386. 28800774 10.1186/s13071-017-2307-1 PMC5553711

[60] Di Francesco CE, Smoglica C, Paoletti B, Angelucci S, Innocenti M, Antonucci A, et al. Detection of selected pathogens in Apennine wolf (*Canis lupus italicus*) by a non-invasive GPS-based telemetry sampling of two packs from Majella National Park, Italy. Eur J Wildl Res. 2019;65:84. 32214950 10.1007/s10344-019-1326-y PMC7088344

[61] Tieri EE, Saletti MA, D’Angelo AR, Parisciani G, Pelini S, Cocco A, et al. *Angiostrongylus vasorum* in foxes (*Vulpes vulpes*) and wolves (*Canis lupus italicus*) from Abruzzo region, Italy. Int J Parasitol Parasites Wildl. 2021;15:184–94. 34136344 10.1016/j.ijppaw.2021.05.003 PMC8182381

[62] Martínez-Rondán FJ, de Ruiz Ybáñez MR, López-Beceiro AM, Fidalgo LE, Berriatua E, Lahat L, et al. Cardiopulmonary nematode infections in wild canids: Does the key lie on host-prey-parasite evolution? Res Vet Sci. 2019;126:51–8. 31437776 10.1016/j.rvsc.2019.08.008

[63] Estévez-Sánchez E, Checa R, Montoya A, Barrera JP, López-Beceiro AM, Fidalgo LE, et al. A high prevalence of cardiopulmonary worms detected in the Iberian Wolf (*Canis lupus*): a threat for wild and domestic canids. Animals (Basel). 2022;12:2289. 10.3390/ani12172289. 10.3390/ani12172289 PMC9454501 36078008

[64] Figueiredo A, Oliveira L, de Maira Carvalho L, Fonseca C, Torres RT. Parasite species of the endangered Iberian wolf (*Canis lupus signatus*) and a sympatric widespread carnivore. Int J Parasitol Parasites Wildl. 2016;5:164–7. 27358768 10.1016/j.ijppaw.2016.04.002 PMC4916035

[65] Hermosilla C, Kleinertz S, Silva LM, Hirzmann J, Huber D, Kusak J, et al. Protozoan and helminth parasite fauna of free-living Croatian wild wolves (*Canis lupus*) analyzed by scat collection. Vet Parasitol. 2017;233:14–9. 28043382 10.1016/j.vetpar.2016.11.011

[66] Chao D, Chang K, Huang I. Survival of first-stage larvae of* Angiostrongylus cantonensis* (Nematoda: Metastrongylidae) frozen by a three-step procedure. Bull Inst Zool Acad Sinica 1988; 27:119–25.

[67] Ferdushy T, Hasan MT. Survival of first stage larvae (L1) of *Angiostrongylus vasorum* under various conditions of temperature and humidity. Parasitol Res. 2010;107:1323–7. 20680334 10.1007/s00436-010-2004-x

[68] Ramos RA, Giannelli A, Dantas-Torres F, Brianti E, Otranto D. Survival of first-stage larvae of the cat lungworm *Troglostrongylus brevior* (Strongylida: Crenosomatidae) under different conditions. Exp Parasitol. 2013;135:570–2. 24055217 10.1016/j.exppara.2013.09.009

[69] Bindke JD, Springer A, Böer M, Strube C. Helminth fauna in captive European gray wolves (*Canis lupus lupus*) in Germany. Front Vet Sci. 2017;4:228. 29312968 10.3389/fvets.2017.00228 PMC5743660

[70] Eckert J, Deplazes P. Biological, epidemiological, and clinical aspects of echinococcosis, a zoonosis of increasing concern. Clin Microbiol Rev. 2004;17:107–35. 14726458 10.1128/CMR.17.1.107-135.2004 PMC321468

[71] Gori F, Armua-Fernandez MT, Milanesi P, Serafini M, Magi M, Deplazes P, et al. The occurrence of taeniids of wolves in Liguria (northern Italy). Int J Parasitol Parasites Wildl. 2015;4:252–5. 26042204 10.1016/j.ijppaw.2015.04.005 PMC4443502

[72] Poglayen G, Gori F, Morandi B, Galuppi R, Fabbri E, Caniglia R, et al. Italian wolves (*Canis lupus italicus* Altobello, 1921) and molecular detection of taeniids in the Foreste Casentinesi National Park, Northern Italian Apennines. Int J Parasitol Parasites Wildl. 2017;6:1–7. 28180084 10.1016/j.ijppaw.2017.01.001 PMC5284487

[73] Crotti S, Spina S, Cruciani D, Bonelli P, Felici A, Gavaudan S, et al. Tapeworms detected in wolf populations in Central Italy (Umbria and Marche regions): a long-term study. Int J Parasitol Parasites Wildl. 2023;21:11–6. 37025622 10.1016/j.ijppaw.2023.03.007 PMC10070192

[74] Crotti S, Brustenga L, Cruciani D, Bonelli P, D’Avino N, Felici A, et al. Molecular screening of *Echinococcus* spp. and other cestodes in wild carnivores from Central Italy. Vet Sci. 2023;10:318. 37235401 10.3390/vetsci10050318 PMC10221036

